# Promoting Research Excellence in Down Syndrome: Proceedings of the 5th International Conference of the Trisomy 21 Research Society

**DOI:** 10.1007/s12017-026-08921-0

**Published:** 2026-06-06

**Authors:** Fabio Di Domenico, Marzia Perluigi, Antonella Tramutola, Maria Giulia Bacalini, Daniella Balduino Victorino, Isabel Barroeta, Renata Bartesaghi, Angelo Carfi, Maria Carmona-Iragui, Ann D. Cohen, Alberto C. S. Costa, Floriana Costanzo, Mara Dierssen, Lauren Dunn, Marco Emili, Edward J. Evans, Clíona Farrell, Lisi Flores-Aguilar, Abi Fukami-Gartner, Sandra Gimenez, Ann-Charlotte Granholm, Thessa Hilgenkamp, Antonella Izzo, Sharon J. Krinsky-McHale, Laura del Hoyo Soriano, Laurent Meijer, Bill Mobley, Ivan P. Moskowitz, Dean Nizetic, Jonathan Pierce, Lauren T. Ptomey, Angela L. Rachubinski, Anne-Sophie Rebillat, Randall Roper, Jonathan D. Santoro, Brian G. Skotko, Fiorenza Stagni, Andre Strydom, Louis Valay, Shahid Zaman, Bruna Lancia Zampieri, the Trisomy 21 Research Society Executive Board, Jonathan Pierce, Thessa Hilgenkamp, Fiorenza Stagni, Marco Emili, Frances Wiseman, Randall Ropper, María Carmona-Iragui, Anne-Sophie Rebillat, Eugenio Barone, Marzia Perluigi, Brian Skotko, Shahid Zaman, Esteban Rozen, Ann-Charlotte Granholm-Bentley, Renata Bartesaghi, William Mobley, Jean-Maurice Delabar, Mara Dierssen, Roger Reeves, Andre Strydom, Marie-Claude Potier, Eugenio Barone

**Affiliations:** 1 Department of Biochemical Sciences, Sapienza University of Rome, Rome, Italy; 2 Department of Biomedical and Neuromotor Sciences, University of Bologna, Bologna, Italy; 3 IRCCS Istituto delle Scienze Neurologiche di Bologna, Bologna, Italy; 4 Institut du Cerveau - Paris Brain Institute - ICM, CNRS, APHP, Hôpital de La Pitié Salpêtrière, Inserm, Sorbonne Université, Paris, France; 5 Sant Pau Memory Unit, Department of Neurology, Hospital de la Santa Creu i Sant Pau, Biomedical Research Institute Sant Pau, Barcelona, Spain; 6 Center of Biomedical Investigation Network for Neurodegenerative Diseases (CIBERNED), Madrid, Spain; 7 Fondazione Policlinico Universitario Agostino Gemelli IRCCS, Rome, Italy; 8 Barcelona Down Medical Center, Fundacio Catalana Sindrome de Down, Barcelona, Spain; 9 Department of Psychiatry, University of Pittsburgh School of Medicine, Pittsburgh, PA USA; 10 Department of Psychiatry, Case Western Reserve University, Cleveland, OH USA; 11 Child and Adolescent Neuropsychiatry Unit, Bambino Gesù Children’s Hospital, Research Hospital, Rome, Italy; 12 Centre for Genomic Regulation (CRG), The Barcelona Institute of Science and Technology, Barcelona, Spain; 13 Universitat Pompeu Fabra (UPF), Barcelona, Spain; 14 Biomedical Research Networking Center for Rare Diseases (CIBERER), Barcelona, Spain; 15 Linda Crnic Institute for Down Syndrome, University of Colorado Anschutz, Aurora, CO USA; 16 Department of Biochemistry and Molecular Genetics, University of Colorado Anschutz, Aurora, CO USA; 17 UK Dementia Research Institute at University College London, UCL Queen Square Institute of Neurology, London, UK; 18 Department of Pathology and Laboratory Medicine, University of California Irvine, Irvine, CA USA; 19 Department of Early Life Imaging, School of Biomedical Engineering and Imaging Sciences, King’s College London, London, UK; 20 Department of Forensic and Neurodevelopmental Sciences, Institute of Psychiatry, Psychology and Neuroscience, King’s College London, London, UK; 21 Alzheimer Down Unit, Barcelona, Spain; 22 Multidisciplinary Sleep Unit, Hospital de la Santa Creu i Sant Pau, Institut d’Investigacio Biomedica Sant Pau (IIB SANT PAU), Barcelona, Spain; 23 Department of Neurosurgery, University of Colorado Anschutz Medical Campus, Aurora, CO USA; 24 Department of Physical Therapy, University of Nevada, Las Vegas, NV USA; 25 Department of Molecular Medicine and Medical Biotechnology, University of Naples ¨Federico II¨, Naples, Italy; 26 New York State Institute for Basic Research in Developmental Disabilities, Staten Island, NY USA; 27 Perha Pharmaceuticals, Roscoff, France; 28 Department of Neurosciences, University of California San Diego, La Jolla, CA USA; 29 Departments of Pediatrics, Pathology, and Human Genetics, University of Chicago, Chicago, IL USA; 30 Faculty of Medicine and Dentistry, Blizard Institute, Queen Mary University of London, London, UK; 31 Department of Neuroscience, University of Texas at Austin, Austin,, TX USA; 32 Department of Internal Medicine, University of Kansas Medical Center, Kansas, KS USA; 33 Institut Jerome Lejeune, Paris, France; 34 Indiana University Indianapolis, Indianapolis, IN USA; 35 Division of Neurology, Children’s Hospital Los Angeles, Los Angeles, CA USA; 36 Department of Neurology, Keck School of Medicine of USC, Los Angeles, CA USA; 37 Down Syndrome Program, Division of Medical Genetics and Metabolism, Department of Pediatrics, Massachusetts General Hospital, Boston, MA USA; 38 Department of Pediatrics, Harvard Medical School, Boston, MA USA; 39 Department for Life Quality Studies, University of Bologna, Rimini, Italy; 40 Institute of Psychiatry, Psychology & Neuroscience, King’s College London; and South London and the Maudsley NHS Foundation Trust, London, UK; 41 Department of Psychiatry, University of Cambridge, Cambridge, UK; 42 Department of Genetics and Evolutionary Biology, Institute of Biosciences, University of São Paulo, São Paulo, , São Paulo, Brazil

**Keywords:** Down syndrome, Trisomy 21, Genomics, Molecular mechanisms, Cognition, Neurodevelopment, Aging, Alzheimer’s disease, Biomarkers, Therapeutics

## Abstract

Down syndrome (DS), or trisomy 21 (T21), represents the most common genetic cause of intellectual disability worldwide and is associated with a wide range of medical, developmental, and neurodegenerative conditions, including a universal predisposition to early-onset Alzheimer’s disease (AD). Since its establishment in 2014, the Trisomy 21 Research Society (T21RS) has provided a global forum for advancing DS research across disciplines and promoting translational efforts to improve health and quality of life. Every two years, T21RS hosts an international scientific meeting that brings together researchers, clinicians, self-advocates, families, and industry stakeholders. In 2024, the 5th T21RS International Conference was held in Rome, Italy, from June 5 to 8, under the theme “Promoting Research Excellence in Down Syndrome.” The meeting brought together about 500 scientists from 26 countries across five continents, and more than 900 attendees overall, including families and caregivers. The scientific program featured 5 keynote lectures, 2 satellite meetings, 17 symposia, 7 nano symposia, 2 workshops, and 1 industry-focused session, totaling more than 150 oral presentations. More than 230 abstracts were presented as posters. The conference covered research across the lifespan of individuals with DS, spanning genomic and epigenetic regulation, molecular and cellular mechanisms, preclinical and experimental models, cognition and behavior, neurodevelopment, aging and neurodegeneration, co-occurring medical conditions, and therapeutic interventions. Dedicated sessions focused on capacity-building in DS research and societal engagement were established. Significantly, T21RS promoted inclusivity by supporting 60 young investigator fellowships, providing childcare awards, and organizing a two-day program for families and caregivers in collaboration with Italian DS associations. This proceeding summarizes the main scientific highlights of the 5th T21RS International Conference, reflecting the latest advances in DS biology, clinical research, biomarker development, and therapeutic innovation.

## Introduction

**Fig. 1:**
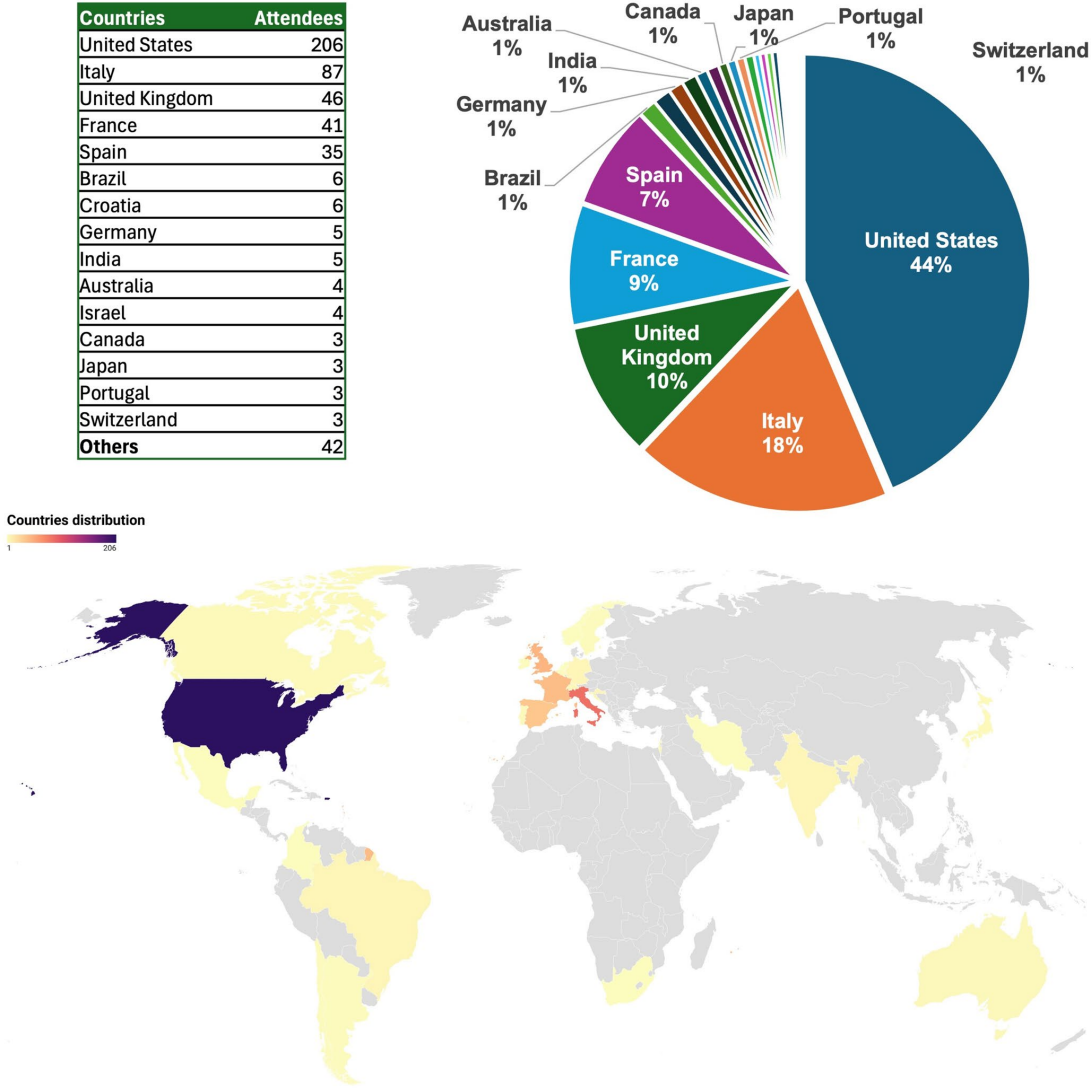
Number of attendees at the 5th International Conference of the Trisomy 21 Research Society and their distribution across countries

The Trisomy 21 Research Society (T21RS) was founded in 2014 with the mission of promoting high-quality, collaborative, and translational research on Down syndrome (DS). Since then, T21RS has grown into a leading international network of scientists, clinicians, and stakeholders dedicated to improving the health and quality of life of people with DS across their lifespan. Every two years, the Society organizes an international meeting that provides a unique platform to present cutting-edge science, foster collaborations, and engage with families and self-advocates (Delabar et al., 2016; Flores-Aguilar et al., 2025; Reeves et al., 2019). The 5th T21RS International Conference was held in Rome, Italy, from June 5 to 8, 2024, under the motto “Promoting Research Excellence in Down Syndrome.” This edition, which celebrated the 10th anniversary of the biennial meeting, marked the largest T21RS conference to date, with 500 scientists representing 26 countries and more than 900 attendees overall, including families, caregivers, and industry representatives (Fig. 1).

Celebrating the 10th anniversary of the Trisomy 21 Research Society, the Rome meeting marked a milestone in the field’s evolution. The scale of participation, the breadth of scientific themes, and the strong engagement of families, institutions, and industry partners reflected T21RS’s transition from an emerging research network to a structured international infrastructure for DS research, with increasing translational and societal impact. The scientific program was extensive, featuring 5 keynote lectures, 2 satellite meetings, 17 symposia, 7 nanosymposia, 2 workshops, and 1 industry session dedicated to clinical trials in DS, totaling more than 150 oral presentations. About 230 abstracts were presented as poster contributions, spanning multiple domains from basic mechanisms to clinical translation.

In addition to the scientific program, the conference prioritized inclusivity and capacity building, awarding 60 fellowships to support young investigators, providing childcare awards, and offering dedicated facilities to enable researchers with families to participate. A two-day family program, organized in partnership with Italian associations (AIPD, Coordown, and ANFASS), engaged over 400 family members through seminars, practical workshops, and medical consultations, underscoring the conference’s societal impact.

The following sections provide a detailed overview of the scientific highlights of the Rome meeting, organized by major thematic areas: Experimental Models; Genomic and Epigenetic Mechanisms; Molecular and Cellular Mechanisms; Cognition and Behavior; Neurodegeneration and Aging; Neurodevelopment; Co-occurring Illnesses; Therapeutic Interventions; Diagnosis and Evaluation; and DS International Capacity Building (Table 1).

**Table 1:** Symposia within each thematic area and participating speakers

Topic	Symposium title	Speakers
**Genomic and epigenetic mechanisms**	***Beyond the gene-dosage hypothesis in trisomy 21***	***Stylianos Antonarakis (Switzerland); Giuseppe Testa (Italy); Cesar Sierra (Spain); María Martínez de Lagrán (Spain)***
	***Is gene therapy a perspective in Down syndrome?***	***Alberto Auricchio (Italy); Antonella Izzo (Italy); Yasuji Kitabatake (Japan); Stefan Pinter (USA)***
**Molecular and Cellular Mechanisms**	***Heart development and disease in Down syndrome***	***Andreia Bernardo (Portugal); Roger Reeves (USA); Victor Tybulewicz (UK); Ivan Moskowitz (USA)***
	***Understanding progeroid biology of Down syndrome throughout lifetime***	***Maria Giulia Bacalini (Italy); Bernard Khor (USA); Dean Nižetić (UK); Arturo Bujarrabal-Dueso (Germany)***
**Experimental models**	***Trisomy 21 research models strengths and limitations (Preclinical committee)***	***Jon Pierce***,*** USA; Summer Thyme***,*** USA; Aoife Murray***,*** UK; Eugene Yu***,*** USA; Sylvia Pérez***,*** USA***
	***Rodent models of Down syndrome: Present and future considerations***	***Randall Roper (USA); Tarik Haydar (USA); Mara Dierssen (Spain); Yann Herault (France)***
**Cognition and Behaviour**	***Cognitive outcome measures in Down syndrome across the lifespan***	***Anna Esbensen (USA); Leonard Abbeduto (USA); Hailey Van Vorce (USA); Laura del Hoyo Soriano (Spain)***
	***Clinical and neurodiagnostic biomarkers in Down syndrome regression disorder: diagnostic and therapeutic Implications***	***Cathy Franklin (Australia); Diego Real de Asua (Spain); Stephanie Santoro (USA); Jonathan Santoro (USA)***
**Neurodegeneration and Ageing**	***The relationship between sleep***,*** epilepsy and cognition in Down syndrome***	***Floriana Costanzo (Italy); Sandra Giménez (Spain); María Carmona-Iragui (Spain); Fedele Dono (Italy); Charles Hoeffer (USA); Chiara Berteotti (USA)***
	***Understanding Alzheimer’s disease in Down syndrome using omics approaches on human biosamples***	***Mark Mapstone (USA); Vivek Swarup (USA); Cliona Farrell (UK); Olivia Belbin (Spain)***
	***Interplay between Alzheimer’s disease and cerebrovascular pathology in individuals with Down syndrome***	***Louis Valay (France); Lisi Flores-Aguilar (USA); Adam M. Brickman (USA); Donna Wilcock (USA)***
**Co-occurring Illnesses**	***Metabolic health in persons with Down syndrome: causes***,*** consequences***,*** and treatment***	***Leda Bianchi (UK); Lauren Ptomey***,*** (USA); Victoria Fleming (USA); Amy Bodde (USA)***
	***Hematopoietic dysregulation inpeople with Down syndrome***	***Irene Roberts (UK); Edward Evans Jr. (USA); Shai Izraeli (Israel); Karen Rabin (USA)***
	***The role of DYRK1A in Down syndrome and related comorbidities: what is new?***	***Daniella Balduino Victorino (France); Julien Caillette (France); Stella Chou (USA); Hidehiro Suginobe (Japan); Jamiley Movassat (France); Laurent Meijer (France)***
	***Liver dysfunction in Down syndrome***	***Lauren Dunn (USA); G. William Wong (USA); April Adams (USA); Diletta Valentini (Italy)***
**Therapeutic intervention**	***Immunomodulatory therapies in Down syndrome***	***Angela Rachubinski (USA); Jonathan Santoro (USA); Jessica Bloom (USA); Joaquin Espinosa (USA)***
	***Industry session Panel 1: Ongoing clinical trials in Down syndrome***	***Pier-Vincenzo Piazza (AELIS Pharma); Laurent Meijer***,*** (Perha Pharmaceuticals); Laura Cancedda (IMA Therapeutics)***
	***Industry session Panel 2: Treatment of Alzheimer’s disease in Down Syndrome***	***Andrea Pfeifer (AC Immune); James Hendrix (Lilly); Patrick Kesslak (Alzheon)***
**Advancing care**,** Diagnosis and Evaluation**	***Healthy lifestyles***,*** clinical***,*** evaluations and cognitive decline in Down syndrome people (Clinical committee)***	***Nicolas M. Oreskovic (USA); Brian Skotko***,*** (USA); Stephanie Santoro (USA); Sigan L. Hartley (USA); Shahid Zaman (UK); André Strydom (UK)***
	***From fetus to old age: advanced neuroimaging biomarkers and their predictive power for cognitive outcomes across the lifespan in Down syndrome***	***Abi Fukami-Gartner (USA); Nancy Raitano Lee’s (USA); Fedal Saini (UK); Herminia Diana Rosas (USA)***
	***Recent advances in understanding Alzheimer’s disease in adults with Down syndrome: Identifying clinical progression and biomarker accumulation***	***Sharon J. Krinsky-Mchale (USA); Laura Videla Toro (Spain); Sigan L. Hartley (USA); Patrick Lao (USA)***
**DS international capacity building**	***Workshop 1: Developing data ecosystems for collaborative Down syndrome research in the age of artificial intelligence***	***Angelo Carfi (Italy); Huiqing Li (USA); Joaquín Espinosa (USA); Micah Donovan (USA); Giuseppe Ramacieri (Italy); Pietro Lió (UK)***
	***Workshop 2: International research programmes to tackle co-occurring conditions in Down syndrome: barriers and facilitators***	***André Strydom (UK); Alberto Bacci; Marie-Claude Potier (France); Yann Herault (France); Ben Handen and Elizabeth Head (USA); Rafael de la Torre (Spain); Orestes Forlenza (Brazil)***

## Experimental Models

Modeling DS in preclinical systems is fundamental to dissecting the genetic, cellular, and molecular underpinnings of trisomy 21 (T21) and to evaluating potential therapeutic interventions.

### Trisomy 21 Research Models Strengths and Limitations

The Preclinical Committee of T21RS organized a pre-meeting symposium highlighting the strengths and limitations of model systems ranging from invertebrates to vertebrates and human-derived cellular models. **Jon Pierce** (USA) presented *Caenorhabditis elegans* as a tractable system for systematic knockout and overexpression screening of Hsa21 genes (Nordquist et al., 2018). His work revealed understudied DS-related genes involved in synaptic transmission and axon guidance and identified expression ceilings for duplication-sensitive genes conserved across worm and human. **Summer Thyme** (USA) discussed *Danio rerio* (zebrafish) as an efficient vertebrate model for studying T21 gene orthologs. Zebrafish offer high gene conservation, external development, optical transparency, and throughput advantages over murine systems, providing unique opportunities for functional studies. **Aoife Murray** (UK) focused on induced pluripotent stem cells (iPSCs) and organoids, noting that neural organoids have thus far been most widely used in DS research. She emphasized the relevance of new international standards for stem cell research and highlighted how brain organoids can recapitulate early neurodevelopmental phenotypes of T21. **Eugene Yu** (USA) presented the latest advances in mouse and rat models of DS, describing how novel genetic engineering strategies are improving the dissection of gene dosage contributions and the effects of an additional chromosome beyond dosage alone. These new models allow more precise mapping of genotype–phenotype relationships (Duchon et al., 2022; Kazuki et al., 2022; Xing et al., 2023). **Sylvia Pérez (USA)** stressed the importance of human postmortem DS brain tissue, considered the gold standard for neuropathogenesis studies. She described the creation of the *Down Syndrome Biobank Consortium (DSBC)*, a worldwide collaborative effort to collect and disseminate high-quality postmortem brain samples from individuals with DS for the research community (Aldecoa et al., 2024). The session concluded with a lively panel discussion that emphasized the complementary nature of animal, cellular, and organoid models. Speakers also highlighted the urgent need for accessible, standardized DS biobank resources to accelerate translational research.

### Rodent Models of Down Syndrome

Rodent models have been indispensable in characterizing the genotype-phenotype relationship in DS and for proposing etiology-based therapies for DS-associated medical conditions. There are many DS rodent models, and careful use of these is critical to advancing our knowledge of DS. In a dedicated symposium, speakers reviewed the latest advances in rodent modeling of DS. **Randall Roper** (USA) discussed how DS mouse models recapitulate some sex-specific skeletal and neurodevelopmental phenotypes observed in individuals with DS (Hawley et al., 2024; LaCombe et al., 2024). He ascertained that understanding the etiological differences between the sexes will be important for sex-specific treatments to improve the lives of individuals with DS. **Tarik Haydar** (USA) presented data showing differences in prenatal neurodevelopmental phenotypes between the new TcMAC21 mouse model and humans and compared developmental changes with those observed in humans. He showed that changes in the activity of specific precursor cell types led to altered neuronal composition in superficial neocortical layers, providing a temporal understanding of what may occur in humans. **Mara Dierssen** (Spain) highlighted the importance of social and environmental factors in shaping behavioral phenotypes in DS mouse models (Fructuoso et al., 2023; Sierra et al., 2021; Wahl et al., 2024). She emphasized that small environmental changes, such as diet, the number of trisomic mice per cage, or the experimenter’s experience or sex, can significantly alter DS-associated phenotypes in mice, and that careful consideration is needed to minimize environmental influences on phenotypic variation and to translate these behaviors to humans. **Yann Herault** (France) presented state-of-the-art genetic manipulations in both mouse and rat models of DS to define the gene-phenotype relationship in DS. He advocated using and developing new and improved mouse models to more accurately reflect the triplicated genes and varied phenotypes observed in individuals with DS (Duchon et al., 2022; Lanzillotta et al., 2024). Moreover, treatment should be tested on several models, including the most complete DS model. These novel insights highlighted essential considerations for modeling DS in rodents. Together, these contributions underscored the indispensable role of experimental models in unraveling the pathophysiology of DS. They highlighted the importance of cross-species approaches, standardized practices, and biobanking resources to ensure reproducibility and translational relevance.

A dedicated nanosymposia focused on understanding the mechanisms underlying DS-AD disease in rodent models. At the nanosymposia, speakers discussed the resources available at the Jackson Laboratory and their differential importance in studies of obesity, the neuroimmune system, and AD-like neurodegeneration.

## Genomic and Epigenetic Mechanisms

Genomic and epigenetic regulation represent a rapidly evolving field in DS research. Beyond the classical gene-dosage hypothesis, new data demonstrate that trisomy 21 alters genome architecture, chromatin accessibility, and epigenomic stability, with consequences extending beyond Hsa21. These findings open new perspectives for understanding DS phenotypes and exploring targeted therapies.

### Beyond the Gene dosage Hypothesis

A symposium was dedicated to evaluating triggering factors and mechanisms beyond the gene-dosage hypothesis in trisomy 21. Indeed, although the chromosomal basis of DS has been known for nearly a century, its molecular consequences remain incompletely understood. Recent single-cell transcriptomic approaches have revealed highly cell-type–specific alterations in gene expression, challenging the simplistic view of uniform dosage effects. **Stylianos Antonarakis** (Switzerland) demonstrated that trisomy 21 causes genome-wide disruption of gene expression through modified chromatin remodeling and nuclear organization (Letourneau et al., 2014). **Giuseppe Testa** (Italy) presented his work focused on “Gene dosage in four dimensions,” utilizing spatiotemporal deconvolution to understand how copy number variations (CNVs) affect human neurodevelopment (Zanella et al., 2019). **Cesar Sierra (Spain)** presented data from trisomic mouse hippocampus (Ts65Dn and Dp16), showing global reductions in histone H3 acetylation and decreased chromatin accessibility at gene promoters. Remarkably, treatment with the histone deacetylase inhibitor SAHA restored regular gene expression and rescued memory deficits, implicating histone acetylation as a therapeutic target (Sierra et al., 2024). **María Martínez de Lagrán (Spain**) highlighted increased retrotransposon activity, particularly LINE-1 elements, in trisomic models. Treatment with lamivudine, a reverse transcriptase inhibitor, improved cognitive performance, normalized expression of several trisomic genes, and reduced LINE-1 dysregulation, suggesting retrotransposition as a contributor to DS neuropathology (Martinez de Lagran et al., 2022). Collectively, these results expand the classical dosage model, pointing toward chromatin dynamics, genome architecture, and transposable element activity as key drivers of DS phenotypes.

### Gene Therapies

A further landmark symposium explored the potential of gene therapy and genome editing for correcting dosage-sensitive pathways in DS. **Alberto Auricchio** (Italy) presented novel gene therapy strategies, including dual adeno-associated viral (AAV) vectors capable of carrying larger genetic material or editing genomic loci in vivo, overcoming traditional vector limitations. Proof-of-concept studies in animal models demonstrated feasibility (Esposito et al., 2024). **Antonella Izzo** (Italy) presented her studies concerning the targeting of dosage-sensitive Hsa21 genes. Her work showed that modulating *NRIP1*, *RUNX1*, and *SYNJ1* reversed mitochondrial, extracellular matrix, and early endosome dysfunction in preclinical studies (Mollo et al., 2021, 2022; De Rosa et al., 2022). Advances in CRISPR-Cas9 technology are now enabling precise correction of these pathways. **Yasuji Kitabatake** (Japan) presented on how overexpression of DYRK1A increased apoptosis in DS astrocytes (Suginobe et al., 2023). CRISPR-Cas3–based dosage correction successfully reversed neuronal cell death, suggesting a promising therapeutic avenue. **Stefan Pinter** (USA) presented his work demonstrating that silencing the extra chromosome 21 via XIST could normalize neural differentiation and reduce aberrant astrocyte formation, even in post-mitotic neurons (Bansal et al., 2024). Altogether, these findings demonstrate the growing feasibility of gene- and genome-based therapies in DS, while underscoring the need for rigorous preclinical validation and ethical consideration before translation to human trials. Selected young investigators discussed their data at a Nano Symposium dedicated to genomic and epigenetic mechanisms, therapeutic approaches, and diagnosis. This session provided novel perspectives on epigenetic regulation and its translational implications. Presentations highlighted the integration of multi-omic approaches to characterize epigenomic landscapes in DS and their potential as biomarkers or therapeutic targets. Discussions emphasized the importance of chromatin regulation and DNA methylation profiling in predicting developmental trajectories and susceptibility to comorbidities.

## Molecular and Cellular Mechanisms

Understanding the molecular and cellular basis of trisomy 21 (T21) is central to linking gene dosage to the multiple organ-specific pathologies of DS. The conference included symposia and nano symposia dedicated to cardiovascular development, progeroid biology, immune dysregulation, and cellular signaling pathways. Congenital heart disease (CHD) is the most common birth defect associated with DS, occurring in ~ 50% of cases and representing a 2000-fold increased risk compared to the general population.

### Heart development and Defects

A symposium addressed mechanistic insights into DS-specific cardiovascular organogenesis defects using new in vitro and in vivo models. **Andreia Bernardo** (Portugal) presented data from human induced pluripotent stem cells (iPSCs) modeling cardiomyocyte development in DS, revealing altered maturation trajectories and gene regulatory networks (Cranley et al. 2025). **Roger Reeves** (USA) discussed how T21 affects cardiac progenitor development, focusing on altered signaling pathways that drive abnormal lineage commitment (H. Li et al. 2012). **Victor Tybulewicz** (UK) described genetic mapping studies in mouse models that identify chromosomal regions that must be triplicated to cause CHD, providing a framework for dissecting the genetic drivers of DS-associated cardiac defects (Lana-Elola et al. 2016, 2024). **Ivan Moskowitz** (USA) highlighted the disruption of Hedgehog signaling in T21 iPSC-derived cardiac lineages, linking altered developmental signaling to congenital defects (Rowton et al. 2022). Together, these presentations advanced mechanistic understanding of DS-associated CHD, identifying gene networks and pathways with potential as therapeutic targets.

### Progeroid Biology

It is widely recognized that DS is associated with an increased prevalence of several hallmarks of aging (Zigman, 2013). The symposium addressed the peculiar progeroid phenotype of people with DS by analyzing biomarkers of aging (epigenetic, immunologic, glycomic) and discussing their dynamic regulation across the lifespan, their association with comorbidities, and possible underlying mechanisms. **Maria Giulia Bacalini** (Italy) showed that blood DNA methylation alterations are established early and tend to remain stable throughout life in people with DS, without evident acceleration. The comparison of changes in DNA methylation during physiological aging suggested that the epigenetic aging of people with DS is embedded in the developmental alterations associated with trisomy 21. By performing a deep immunophenotyping of individuals with DS of different ages, **Bernard Khor** (USA) also demonstrated that advanced immune aging in people with DS begins from childhood and persists across the lifespan (Lambert et al., 2022). Furthermore, examining responses to SARS-CoV-2 vaccination revealed distinct defects in antigen-specific versus non-antigen-specific B cells in people with DS, illuminating functional pathways of inflammaging dysregulated in DS. In line with the first two talks, **Dean Nižetić** (UK) showed that biological age, as measured by plasma IgG-glycans, increased from childhood in DS and remained constant throughout the lifespan, independent of co-morbidities. Importantly, Nižetić demonstrated that the increased biological age was dependent on trisomy of the *DYRK1A* gene, which leads to excess unrepaired DNA damage and, in turn, drives a laminopathy-associated progeroid state (Murray et al., 2023). Finally, **Arturo Bujarrabal-Dueso** (Germany) provided new insights into the role of DYRK1A in regulating the expression of multiple genes involved in the DNA damage response in somatic tissues (Bujarrabal-Dueso et al., 2023). Chemical inhibition of DYRK1A in human cells enhanced DNA damage resistance and genomic stability, suggesting it as a potential pharmacological target for alleviating the progeroid phenotype of DS.

A nano symposium was further dedicated to novel mechanistic studies of T21 cellular biology, focusing on mitochondrial metabolism, intracellular trafficking, and immune regulation. Talks highlighted how trisomy-sensitive pathways alter neuronal differentiation, immune responses, and cellular homeostasis, providing insights into candidate therapeutic targets.

## Cognition and Behavior

Cognition and behavior are central aspects of DS research, as intellectual disability, language difficulties, and behavioral alterations significantly impact quality of life. The conference featured dedicated sessions on cognitive outcome measures, language development, novel neuropsychological tools, and Down Syndrome Regression Disorder (DSRD).

### Cognitive Outcome Measures

A dedicated symposium explored essential tools for assessing cognitive outcomes in DS across the lifespan, with implications for research and interventions. **Anna Esbensen** (USA) opened with a study on normative changes in executive functioning in youth with DS ages 6–17 years, analyzing behavioral, emotional, and cognitive regulation in 104 participants over 12 months (Waschl et al., 2023). This work established baseline developmental trajectories, enabling the interpretation of genuine changes during interventions. **Leonard Abbeduto** (USA) presented data on the Expressive Language Sampling (ELS) procedure, a validated measure of language intervention outcomes in individuals with DS, ages 6–23 (Abbeduto et al., 2023; Thurman et al., 2021). By collecting naturalistic language samples under scripted and structured conditions, ELS has been demonstrated to yield measures with strong reliability, validity, and sensitivity to change, making it highly suitable for studying expressive language development in this population, including within treatment studies. **Hailey Van Vorce** (USA), under the mentorship of Jamie Edgin (USA), presented data on the digital Arizona Memory Assessment for Preschoolers and Special Populations (A-MAP), which assesses neuropsychological functioning through verbal and executive functioning memory tasks. A-MAP has been validated in 152 children with different developmental disabilities, including those with DS. Positive results on reliability, practice effects, and feasibility for remote administration were presented, suggesting its applicability to clinical trials and neuroimaging studies. **Laura del Hoyo Soriano** (Spain) concluded with normative cognitive data for over 600 adults with DS, focusing on early AD symptom detection. Using the DABNI cohort, cognitive performance across intellectual disability levels and clinical diagnoses (e.g., asymptomatic, prodromal AD, and dementia AD) is presented, and cutoff points are established with AUCs on key tools (Benejam et al., 2020; Hoyo et al., 2015) for effective AD screening in the DS population. Collectively, these presentations deepen understanding of DS cognitive trajectories, supporting precise assessments and effective interventions tailored to developmental stages.

### DS Regression Disorder

Over the past five years, significant progress has been made in defining DS Regression Disorder (DSRD), a rare but severe condition characterized by sudden neuropsychiatric decline in adolescents and young adults with DS. Speakers at the dedicated symposium emphasized that the newly established diagnostic criteria achieve > 95% sensitivity and specificity, enabling reliable identification of affected individuals. **Cathy Franklin** (Australia) reviewed catatonia, one of the most complex features of DSRD, with particular attention to the benefits and limitations of existing catatonia constructs and to how these can be applied to individuals with intellectual disability and other neurodevelopmental disorders (J. D. Santoro et al. 2022a, b; J. D. Santoro et al., 2023). Further, she reported on the clinical complexities of catatonia, focusing on adapting diagnostic constructs from intellectual disability and neurodevelopmental disorders. **Diego Real de Asua** (Spain) and **Stephanie Santoro** (USA) presented their work on differential diagnosis with autism spectrum disorder and AD, highlighting the importance of multidisciplinary evaluation (Rosso et al., 2020; J. D. Santoro et al. 2022a, b). Neurodiagnostic (EEG, MRI, CSF analysis) was instrumental in ruling in/out DSRD and in predicting therapeutic responses. **Jonathan Santoro** (USA) demonstrated that neuroimaging abnormalities were associated with an > 8-fold increased likelihood of response to immunotherapy (J. D. Santoro et al. 2024a; J. D. Santoro et al. 2024b; S. L. Santoro et al. 2022a, b). Conclusions emphasized that DSRD is entering a new era of rapid advances in diagnosis and treatment, with neurodiagnostic biomarkers offering a roadmap to individualized therapeutic strategies.

## Neurodegeneration and Ageing

Neurodegeneration represents one of the most critical challenges for individuals with DS. By age 40, virtually all individuals with DS exhibit AD neuropathology, and many progress to dementia in their 50s. The conference featured symposia addressing the interplay between sleep, epilepsy, cognition, AD pathophysiology, and cerebrovascular pathology, supported by new multi-omic studies.

### Sleep, Epilepsy, and Cognition

Sleep is a primary concern for individuals with DS because around half of them have obstructive sleep apnea (OSA), and up to approximately 70% of children with DS experience behavioral sleep problems (Shott et al., 2006). Given the impact of sleep on attention and cognitive abilities, poor sleep in DS can exacerbate DS-related cognitive deficits. Individuals with DS are at very high risk of developing symptoms of AD in early adulthood, a risk that sleep disturbances may exacerbate (Ju et al., 2014). Many individuals with DS experience epilepsy disorders, which may enhance cognitive decline and induce sleep fragmentation. Thus, early sleep disturbances, AD development, and epilepsy in people with DS create a reinforcing triad of conditions. Based on human and translational animal research, a dedicated symposium provided novel viewpoints about the impact of sleep disorders and epilepsy on cognition and AD development in DS. **Floriana Costanzo** (Italy) presented her work showing that sleep alterations are detectable in infants and adolescents with DS and correlate with impaired mental development (Fuca et al., 2021). **Sandra Giménez** (Spain) reported on studies in adults with DS, in which polysomnography data revealed associations between OSA and elevated AD biomarkers in cerebrospinal fluid and plasma (Gimenez et al., 2021). Works by **María Carmona-Iragui** (Spain) and **Fedele Dono** (Italy) discussed epileptic seizures as additional risk factors that exacerbate cognitive decline, with insights from an ongoing clinical trial targeting seizure prevention in DS-AD adults (Gimenez et al., 2025). **Charles Hoeffer** (USA) and **Chiara Berteotti** (USA) presented their preclinical studies that highlighted the role of *RCAN1* triplication in sleep abnormalities (Dp16 mice) and showed that sleep fragmentation accelerates neuronal loss in AD-vulnerable brain regions (Ts65Dn mice) (Wong et al., 2015; Levenga et al., 2018). This symposium emphasized that early identification and treatment of sleep and epilepsy disorders could mitigate neurodegeneration in DS.

### Omics Approaches for AD Progression

A further session explored multi-omic approaches to dissect AD pathophysiology in DS. Three copies of the APP gene on chromosome 21 trigger early amyloid-β (Aβ) accumulation in the brain, leading to the development of dementia (Fortea et al., 2021). However, growing evidence indicates that other dosage-sensitive genes on chromosome 21 modulate key aspects of AD pathogenesis. Gene and protein networks, as well as metabolic and neuro-immune phenotypes, can vary in their similarities and differences between individuals with DS-AD and those with sporadic AD (Martini et al., 2020; Zammit et al., 2024), but the molecular and cellular mechanisms underlying these differences remain poorly understood. New data from spatial and single-nucleus transcriptomics of human postmortem brain tissue revealed cell-type–specific gene expression changes associated with DS-AD (Miyoshi et al., 2024). Building on previous data from the ABC-DS consortium {Mapstone, 2020 #49}, **Mark Mapstone** (USA) presented fluid-based metabolomic profiling that revealed metabolic disequilibrium during early AD phenoconversion in DS, implicating pathways in lipid and energy metabolism. **Vivek Swarup** (USA) underscored the importance of integrative approaches that employ multiple models of investigation and data modalities to elucidate spatially restricted and cell-type-specific transcriptomic changes across different AD subtypes. **Cliona Farrell** (UK) reported on the comparative proteomics of DS and early-onset AD brains, revealing differences in protein networks, including APOE-associated modules. **Olivia Belbin** (Spain) explored CSF and plasma proteomics using the Olink Inflammation panel, demonstrating that AD progression in DS correlates with elevated inflammatory signatures, supporting the use of biomarkers beyond Aβ and tau. Overall, this symposium, using advanced omics technologies, provided critical new insights into DS-AD mechanisms that may have broader implications for treatment choices and therapy development for people with DS.

### Cerebrovascular Pathology

Individuals with DS display significant cerebrovascular pathology that may be linked to the progression of AD (Valay and Potier 2025). This symposium brought together a panel of scientists investigating cerebrovascular changes in DS. **Louis Valay** (France) examined the role of endothelial cells (ECs) in CAA severity using human iPSC-derived ECs (hiPSC-EC) from DS and APPdup. He found a subset of dysregulated genes that decreased EC permeability in DS and favored it in APPdup. An increase in permeability in APPdup could favor the transport of peripheral Aβ, promoting vascular deposits. Individuals with DS would be protected by having a less permeable blood-brain barrier (BBB). **Lisi Flores-Aguilar** (USA) examined BBB integrity and microvascular morphology at advanced stages of AD in DS (DSAD) using postmortem brain tissue (Aguilar et al. 2023). Compared to non-DS controls, DSAD displayed increased vessel and basement membrane density and vessel length, a higher number of pericytes, and collapsed capillaries. Severe parenchymal fibrin deposition was also observed. These findings suggest that BBB integrity in DSAD is compromised. **Adam M. Brickman** (USA) discussed how cerebrovascular disease neuroimaging markers relate to AD biomarkers and cognitive outcomes in adults with DS enrolled in the ABC-DS study. He reported that vascular lesions emerge at about the same time as Aβ and tau pathology in adults with DS and that neuroinflammation is detectable before neurodegeneration. This suggests that vascular and inflammatory factors, along with AD pathology, influence clinical progression of AD in DS (P. Lao et al. 2024; P. J. Lao et al. 2020; Edwards et al. 2024). **Donna Wilcock** (USA) presented a panel of potential fluid biomarkers for vascular cognitive impairment and dementia (VCID). Placental Growth Factor (PlGF) was the most promising biomarker of all the proposed markers. PlGF levels in plasma and CSF can accurately predict WMH and cognitive impairment in VCID patients (Hinman et al. 2023). Dr. Wilcock is currently assessing the implementation of this biomarker panel in samples from adults with DS from the ABC-DS study.

Further, a nanosymposium chaired by **D. Allan Butterfield** (USA) was dedicated to neurodegeneration and ageing, with presentations aimed at covering the association between fetomaternal transfer during DS pregnancies and cognitive decline, the involvement of hypothalamic structure in AD development, and the relationship between mitochondrial dysfunction (UPRmt) and mental deficits, among others.

## Co-occurring Illnesses

Individuals with DS are predisposed to a wide range of comorbid medical conditions, including immune dysregulation, autoimmune diseases, hematological malignancies, and gastrointestinal disorders. The conference hosted several sessions highlighting advances in understanding these co-occurring illnesses and their mechanistic underpinnings.

### Metabolic Health

Individuals with DS face an increased risk of metabolic conditions, such as obesity and type 2 diabetes, compared to the general population (G. Capone et al. 2020; G. T. Capone et al. 2018; Chicoine et al. 2021). A symposium reviewed the metabolic differences between those with and without DS, the link between metabolic health and AD risk, and effective interventions to improve metabolic health in this population. **Leda Bianchi** (UK) discussed findings from the Go-DS21 study, which indicate that individuals with DS are at risk of cardiometabolic disease, with this risk increasing with higher body mass index (BMI) (Aslam et al. 2022). **Lauren Ptomey** (USA) demonstrated that recently completed trials (Ptomey et al. 2018a, b, 2021) have identified effective strategies for achieving significant weight loss and maintaining it over the long term in adolescents and adults with DS using an enhanced Stop Light Diet. **Victoria Fleming** (USA) reported data from the Alzheimer Biomarker Consortium-Down Syndrome, revealing that increased physical activity is associated with reduced cognitive decline over three years, providing further evidence that engaging in more physical activity may have beneficial effects on AD in adults with DS (Fleming et al. 2021; Pape et al. 2021). **Amy Bodde** (USA) reported on a randomized trial demonstrating that a remotely delivered exercise intervention increased physical activity participation by 43% among adults with DS. Overall, this symposium highlights the availability of intervention strategies to promote weight loss and physical activity in persons with DS. Future research should focus on the impact of these interventions on cardiometabolic disease and AD risk in individuals with DS, as well as mechanisms driving these relationships (e.g., inflammation).

### Liver Dysfunction

A further symposium provided a comprehensive investigation into what is currently known about the impact of trisomy 21 on liver function and metabolic health in individuals with DS. Emerging research in this field highlights the specific metabolic dysfunction these individuals experience, including altered bile acid metabolism, prenatal findings of liver-specific phenotypes, biomarkers, and tests to begin to define potential diagnostic criteria, and the use of mouse models to further elucidate mechanisms of dysfunction in this population. **Lauren Dunn** (USA) presented detailed proteomic and metabolomic analyses from the Human Trisome Project Biobank, revealing dysregulation of pathways consistent with liver dysfunction in individuals with DS (Dunn et al., 2025). **G. William Wong** (USA) presented data on the Dp16 mouse model, which demonstrates substantial liver pathology and fibrosis. Transcriptomic analysis suggested potential mechanisms underlying this dysfunction, emphasizing the need for further mechanistic studies to identify therapeutic targets (Sarver et al., 2023). A complementary study examined metabolic phenotypes in different DS mouse models. The Dp16 model displayed insulin resistance and impaired lipid metabolism, aligning with observations in the DS population. In contrast, TcMAC21 mice exhibited enhanced insulin sensitivity, showing the complexity of metabolic phenotypes across different models. **April Adams** (USA) reported on another aspect of this field, namely prenatal diagnosis of DS-related phenotypes, which continues to pose challenges (Adams et al., 2020). Findings from placental evaluations revealed increased vascular malperfusion and hematologic abnormalities, suggesting a link between abnormal placentation and perinatal liver dysfunction. These insights highlight the importance of prenatal predictors for phenotypic severity, which could guide antenatal surveillance and intervention. Finally, **Diletta Valentini** (Italy) demonstrated that children with DS show a high prevalence of metabolic dysfunction associated with fatty liver disease (MAFLD) (Scoppola et al., 2025). Genetic factors, such as the PNPLA3 variant, and adipocytokine dysregulation, such as TNF-alpha and adiponectin, may contribute to disease pathogenesis (Martini et al., 2022). Together, these studies underscore the urgent need to better understand liver dysfunction in DS across the lifespan and to develop targeted interventions to improve metabolic health.

### DYRK1A and Related Co-morbidities

A specific symposium brought together a multidisciplinary team investigating various DS-associated conditions potentially affected by DYRK1A overdose. **Daniella Balduino Victorino** (France) presented a new mouse model of tau pathology overexpressing DYRK1A. She showed worsening of spatial learning and memory in double-transgenic mice, likely due to neuropathological changes driven by advanced tau pathology. **Julien Caillette** (France) discussed the use of multimodal magnetic resonance imaging (MRI) to assess brain morphology and functional connectivity (FC) in the Dp16 mouse model of DS. He identified widespread morphological alterations using voxel-based morphometry and white matter abnormalities using diffusion tensor imaging (DTI). Resting-state functional MRI demonstrated that DYRK1A inhibition altered FC within memory-related nodes, which aligned with improved performance on behavioral tasks in Dp16 mice. **Stella Chou** (USA) studied DS-related transient abnormal myelopoiesis and myeloid leukemia using isogenic induced pluripotent stem cells (iPSCs) (Sit et al., 2023). Her team found that DYRK1A restrained megakaryocyte proliferation in the context of GATA1 but unexpectedly exacerbated expansion of immature megakaryocytes when GATA1s was exclusively expressed. These findings contrast with murine models, in which DYRK1A overexpression promoted megakaryocyte expansion in both GATA1 and GATA1s, highlighting species-specific effects. **Hidehiro Suginobe** (Japan) used T21-iPSC-derived endothelial cells (ECs) to study pulmonary arterial hypertension (PAH) in DS (Suginobe et al., 2023). He observed increased apoptosis, oxidative stress, and impaired angiogenesis, which improved with DYRK1A inhibition. His findings suggest that DYRK1A overexpression in DS contributes to endothelial dysfunction by upregulating EGR1. **Jamiley Movassat** (France) investigated the effects of DYRK1A inhibition on pancreatic β-cell regeneration in a rodent model of Type 2 diabetes (T2D). DYRK1A inhibitors from the Leucettinib family restored pancreatic β-cell mass and function in T2D rats, supporting DYRK1A as a therapeutic target for T2D (Lindberg et al., 2023). Finally, **Laurent Meijer** (France) presented results on Leucettinib-21, now in a Phase 1 clinical trial for healthy volunteers and adults with DS or AD (Meijer et al., 2024). The trial aims to assess the safety and tolerance of Leucettinib-21, a DYRK1A inhibitor with potential therapeutic benefits for cognitive function.

### Hematopoietic Dysregulation

People with DS have increased risks of hematologic abnormalities and diseases (Hasle et al. 2000). The unique progression of the prenatal GATA1 mutation to acute megakaryoblastic leukemia is well-documented in DS research (Hasle et al. 2008). However, several hematopoietic phenotypes and genetic profiles merit discussion and future investigation. In a symposium, presenters sought to highlight ongoing research into hematopoietic manifestations in people with DS. **Irene Roberts** (UK) began the session by highlighting pre- and postnatal disruptions in the hematopoietic system in people with DS. These phenotypes contribute to the manifestation of a higher prevalence of acute lymphoblastic leukemia (ALL) and acute myeloid leukemia (AML) in people with DS. Additionally, advanced epigenetic aging is observed in newborns with DS (Xu et al. 2022), which may be a cause or effect of their dysregulated hematopoietic system. **Edward Evans Jr.** (USA) presented work on clonal hematopoiesis in people with DS and in the age-matched general population, observing an overrepresentation of TET2 mutations in people with DS, as previously shown in children (Liggett et al. 2021). It is hypothesized that disruptions in hematopoietic differentiation or low cell proliferation may promote TET2 mutations. Future investigations of granulocytes and monocytes in people with DS may shed light on this phenomenon. **Shai Izraeli** (Israel) discussed the importance of the JAK-STAT pathway in the relapse of ALL in people with DS. Through genetic profiling at diagnosis, remission, and relapse, it was determined that CRLF2 rearrangements were initiating mutations, while various secondary activating mutations enabled relapses through JAK-STAT signaling (Schwartzman et al. 2017). This highlights the challenges in applying JAK inhibitors in DS-ALL. **Karen Rabin** (USA) presented current knowledge and ongoing investigations into host susceptibility factors associated with the 20-fold increased risk of developing ALL in children with DS. Rabin discussed somatic features of DS-ALL, as defined by cytogenetics and whole-genome and transcriptome sequencing, germline variants associated with ALL susceptibility, and the interplay between other DS-related phenotypes and ALL susceptibility (Brown et al. 2019; Z. Li et al. 2023).

A nanonsymposia discussed epidemiology, pathophysiology, and clinical symptomology studies, which revealed that individuals with DS were at a higher risk for serious complications and a 10-fold higher mortality rate in cases with SARS-CoV-2 than other people. Speakers discussed the cellular and molecular neuropathology found in developing and aged DS brains and viral pathology in those who succumbed to the SARS-CoV-2 virus.

In conclusion, research on co-occurring illnesses in DS reveals converging mechanisms involving metabolic dysregulation, interferonopathy, and hematopoietic vulnerabilities. Improved understanding of these pathways is critical for developing precision medicine strategies to prevent or mitigate comorbid conditions in DS.

## Therapeutic Interventions

Developing effective therapies for DS requires approaches that target both core cognitive features and co-occurring medical conditions. The conference showcased innovative pharmacological strategies, prenatal interventions, and clinical trials aimed at improving health outcomes across the lifespan.

### Immunomodulatory Therapies

A symposium covered recent advances in the study of immunomodulatory therapies in DS for diverse clinical endpoints. Although it is now well accepted that immune dysregulation is a hallmark of DS across the lifespan, the therapeutic implications remain to be defined (Sullivan et al., 2016). **Angela Rachubinski** (USA) presented results from a clinical trial evaluating the safety and efficacy of a JAK inhibitor across diverse clinical endpoints in DS (Rachubinski et al., 2024). **Jonathan Santoro** (USA) presented results from a clinical trial comparing two immunomodulatory medicines (the JAK inhibitor tofacitinib and intravenous immunoglobulin [IVIG]) with the benzodiazepine lorazepam for DS Regression Disorder (J. D. Santoro et al., 2023). **Jessica Bloom** (USA) presented results from the use of JAK inhibitors and other immunomodulatory agents for the treatment of diverse conditions in children with DS (Jones, 2022). **Joaquin Espinosa** (USA) presented new results demonstrating in utero dysregulation of interferon responses and inflammatory signaling in DS and discussed the therapeutic implications of these findings toward the development of prenatal treatments (Waugh et al., 2023).

### Industry Session

A dedicated industry session provided an in-depth overview of ongoing and planned clinical trials targeting individuals with DS, with a strong emphasis on preventive and disease-modifying strategies for AD, which almost universally develops in this population with age. A central focus of the discussion was the advancement of anti-amyloid and anti-tau therapeutic approaches, reflecting the growing translational momentum to adapt AD treatments specifically to the DS context.

Speakers highlighted the growing importance of biomarker-driven clinical trial design, underscoring how integrating multimodal biomarkers is reshaping therapeutic development. Particular attention was given to neuroimaging markers, plasma-based assays, and cerebrospinal fluid (CSF) biomarkers as tools for early detection, patient stratification, and longitudinal monitoring of disease progression. In parallel, digital cognitive endpoints were discussed as innovative and sensitive measures that can capture subtle cognitive changes in individuals with DS, thereby enhancing trial efficiency and feasibility.

The first panel, dedicated to ongoing clinical trials in DS, featured **Pier-Vincenzo Piazza** (AELIS Farma), **Laurent Meijer** (Perha Pharmaceuticals), and **Laura Cancedda** (IMA Therapeutics). This panel provided an overview of planned or ongoing studies designed to test whether treatments targeted at biological pathways implicated in Down syndrome could improve cognitive functioning.

The second panel focused specifically on the treatment of AD in DS and included **Andrea Pfeifer** (AC Immune), **James Hendrix** (Lilly), and **Patrick Kesslak** (Alzheon). Presentations addressed advances in amyloid-targeting compounds and other emerging disease-modifying approaches. This panel focused on the potential for next-generation treatments for Alzheimer’s disease to tackle the onset of neurodegeneration and dementia in people with Down syndrome, including amyloid vaccines and other means to block the formation of neurotoxic soluble amyloid oligomers.

## Advancing Care, Diagnosis, and Evaluation

The T21RS clinical committee organized a satellite symposium on Healthy lifestyles, clinical evaluations, and cognitive decline in people with DS.

### Advancing Care

The first part of the satellite symposium was dedicated to advancing care for individuals with DS, addressing co-occurring health conditions, lifestyle management, and personalized healthcare solutions (Tsou et al., 2020). **Stephanie Santoro** (USA) presented a Dementia Protocol developed to support adults with DS at heightened risk of AD (Harisinghani et al., 2024; S. L. Santoro et al., 2025). This protocol includes tools for clinical screening, neuropsychological and medical evaluation, mental wellness, and family support. **Nicolas Oreskovic** (USA) discussed strategies to promote healthy lifestyles in individuals with DS, emphasizing weight management, physical activity, sleep care, and dietary guidance (S. L. Santoro et al., 2025; Ptomey et al., 2022). **Brian Skotko** (USA) introduced the “Down syndrome Clinic to You” (DSC2U), an online platform that provides customized health plans for caregivers and primary care physicians, helping to bridge access gaps for those unable to attend specialty clinics (Chung et al. 2021a, b). Together, these approaches aim to enhance long-term health and quality of life for people with DS and their families.

### Diagnosis and Evaluation

In the second part of the satellite symposium, **Sigan L. Hartley** (USA) presented recent cross-sectional and longitudinal research on the cognitive performance of adults with DS, examining age and biomarkers of early AD pathology (Schworer et al., 2024; Hartley et al., 2023). Findings related to the timing of AD pathology and cognitive decline based on the level of intellectual disability were highlighted. The implications of these findings for the design of clinical intervention trials were discussed. **Shahid Zaman** (UK) reported on the spectrum of the development and onset of neurodegeneration in people with DS. Indeed, as the field of biomarkers of AD in DS develops, we are faced with knowing the importance of understanding the distinction and the relationship between disease, illness, and sickness (Fortea et al., 2020). The presentation of signs and symptoms in those with compromised intellectual and adaptive functioning impacts all these aspects, but especially on “health behaviours”. The development and the onset of neurodegeneration in people with DS is a slow and long process (Fortea et al., 2021). The relationships between underlying neuropathological tissue changes (disease) and the clinical manifestations (illness) are, by and large, correlative. The long latency between the presence of pathology and the onset of the signs and symptoms needs to be more clearly characterized. By investigating potential biomarkers, it is hoped that the presymptomatic, prodromal, and dementia stages can be distinguished more reliably, related to the underlying pathology, and less likely to be confused with co-occurring conditions that may mimic these clinical phases of the disease. The action taken by the treating clinician depends on the values of the patient, the clinician, and society (sickness behaviour). **Andre Strydom** (UK) presented on Potential challenges and barriers to implementing recently approved pharmacological strategies to treat AD in the general population and in persons with DS (Strydom et al., 2018). After an almost two-decade hiatus following the approval of memantine, the US Food and Drug Administration (FDA) approved two new pharmacological interventions for the treatment of AD: Aducanumab and Lecanemab. These monoclonal antibodies target different aggregation states of Aβ (Aducanamab was designed to bind primarily to Aβ, and Lecanamab shows greater specificity for Aβ protofibrils). Both of these anti-amyloid antibody therapies are based on the principle that AD dementia is the direct consequence of the production and accumulation of Aβ in the brain (the Amyloid Hypothesis). The exact neurodegenerative mechanism is also hypothesized to occur in AD in persons with DS, who are reported to have higher amyloid loads at much younger ages than those with sporadic AD in the general population (Hillerstrom et al., 2024). In this talk, some of the general shortcomings of the approval trials for Aducanumab and Lecanemab were discussed, and the lack of data on safety in people with Down syndrome, who may be at increased risk for cerebral amyloid angiopathy and amyloid-related imaging abnormalities (ARIA) as a consequence of treatment with monoclonal antibodies. A debate ensued on specific barriers to implementing these therapies in persons with DS. The need for further research to identify potential clinical benefits and risks of these therapies was emphasized.

### Advanced Neuroimaging Biomarkers

A dedicated symposium provided an overview of current neuroimaging biomarkers and their potential to predict neurodevelopmental and neurodegenerative outcomes in DS across the lifespan, from fetal life to old age. **Abi Fukami-Gartner** (USA) presented research on brain development in fetuses (20–35 gestational weeks, GW) and neonates (up to 46 corrected GW) with DS, using specialised in utero and neonatal MRI. His findings included a comprehensive volumetric analysis (Fukami-Gartner et al., 2023) leveraging normative models and novel deep-learning parcellation tools for fetal (Uus et al., 2023) and neonatal brains. He also examined links between these imaging results and neurodevelopmental outcomes from birth to over 5 years, highlighting avenues of research for potential early biomarkers of individual outcomes in DS. **Nancy Raitano Lee’s** (USA) presentation examined atypicalities in the structure (Lee et al., 2016), connectivity (Lee et al., 2020), and function (Csumitta et al., 2022) of the frontal lobe in young individuals with DS (ages 6–24). She shared findings from studies using various imaging modalities, including diffusion MRI, as well as a functional near-infrared spectroscopy (fNIRS) study investigating executive control task performance. Her work offered insights into the neural basis of higher-level cognition in DS. It also highlighted the need for longitudinal investigations into the developmental unfolding of frontal lobe atypicalities and executive dysfunction in DS, with the long-term goal of informing developmentally attuned interventions to support cognitive outcomes. **Fedal Saini** (UK) examined the link between trisomy 21 and neurodegeneration in adults with DS (ages 19–58) (Idris et al., 2023)using diffusion MRI data (Saini et al., 2022) from the LonDownS study. The presentation highlighted advanced diffusion modelling and volumetric assessment using diffusion tensor-based morphometry, revealing insights into the onset of AD neuropathology in DS. Rather than localised changes, volumetric and microstructural alterations affect all major white matter (WM) tracts. While corpus callosum, fornix, and temporal WM changes resemble sporadic AD, frontoparietal tract involvement may be unique to AD in DS. Finally, **Herminia Diana Rosas** (USA) discussed WM alterations in the early stages of Huntington’s disease (Demir & Rosas, 2024; Rosas et al., 2010). Her presentation offered a valuable comparative perspective on the use of MRI as a tool for understanding inherited neurodegenerative conditions, complementing our understanding of AD research in DS.

### Clinical Progression and Biomarker Accumulation

Individuals with DS are the largest genetically defined high-risk population for developing the neuropathological features and clinical symptoms of AD. The discovery of effective treatment and prevention methods is among the highest priorities for current biomedical research. Urgent efforts are needed to characterize the clinical and cognitive progression of DSAD, investigate the utility of biomarkers, and design tailored health plans and clinical trials. A dedicated symposium presented data from two large studies: the Alzheimer Biomarker Consortium—Down syndrome (ABC-DS) and the Down syndrome Alzheimer Barcelona Neuroimaging Initiative (DABNI) (Handen et al., 2025; Videla et al., 2025). **Sharon J. Krinsky-Mchale** (USA) and **Laura Videla Toro** (Spain) showed that both studies have been examining clinical, neuropsychological, and biomarker data and their associations with the development of DSAD. Both have identified objective measures sensitive to the emergence of prodromal AD and the transition to DSAD, the feasibility of conducting single-point cognitive assessments, and the examination of imaging biomarkers that facilitate precise detection of cognitive decline. In general, cross-sectional findings have confirmed that our measures are reliable for this target population and are sensitive to the clinical progression of AD (Fortea et al., 2020; Fu et al., 2025). In addition, **Sigan L. Hartley** (USA) and **Patrick Lao** (USA) highlighted the longitudinal validation of neuropsychological tools and their interactions with select imaging biomarkers, including those beyond Aβ and tau, for use in preventive clinical trials (Hartley et al., 2024; Barry et al., 2025). Current anti-amyloid antibody therapeutics have a vascular safety profile that needs careful attention in this population. Identifying dynamic changes in clinical profiles and biomarkers is essential for understanding the progression of AD from cognitive stability to MCI-DS and from MCI-DS to DS-AD. It will inform targets and safety profiles for the next generation of clinical trials.

In conclusion, diagnosis and evaluation in DS are entering a precision medicine era, with biomarkers, neuroimaging, and digital tools offering unprecedented sensitivity. These advances will be critical for clinical trials and for tailoring interventions to the individual needs of people with DS.

## DS international capacity building

**Workshop 1**: *Developing data ecosystems* for collaborative DS research in the age of artificial intelligence, and focused on the urgent need to build interoperable, large-scale data infrastructures to advance collaborative research in DS. Moderated by **Angelo Carfi** (Italy), the session explored how international data sharing, multi-dimensional datasets, and artificial intelligence (AI) approaches are reshaping research strategies in the field. The workshop opened with **Huiqing Li** (USA), who presented the NIH perspective on international cohort development and data sharing. The talk emphasized the importance of harmonizing longitudinal cohorts across countries through standardized data collection and interoperable infrastructures, supported by a cloud-based data ecosystem in which researchers analyze data within governed workspaces rather than duplicating datasets across local silos. This approach promotes FAIR-aligned data reuse while reducing infrastructure duplication, security risks, and analytic fragmentation. **Joaquín Espinosa** (USA) followed with a presentation on the assembly and analysis of multidimensional datasets within the INCLUDE Data Hub. He described the integration of clinical, genomic, and phenotypic data into a centralized infrastructure designed to facilitate cross-study analyses, support reproducibility, and enable advanced computational modeling. **Micah Donovan** (USA) addressed the application of multi-omics approaches to investigate delayed development and early ageing in DS. By integrating transcriptomic, epigenomic, and proteomic data, the presentation highlighted emerging molecular signatures that may underlie both neurodevelopmental trajectories and accelerated ageing processes characteristic of DS. **Giuseppe Ramacieri** (Italy) presented an Italian pilot study aimed at establishing standard criteria for database development and implementing machine-learning-based approaches. The talk underscored the need for data standardization, quality control frameworks, and scalable analytical pipelines to ensure AI tools can be reliably applied in clinical and translational research settings. The workshop concluded with **Pietro Lió** (UK), who discussed the intersection of AI and medicine through a case study on co-occurring conditions in DS. The presentation illustrated how machine learning algorithms can identify complex patterns of multimorbidity, predict risk trajectories, and inform personalized healthcare strategies.

**Workshop 2**: *International research programmes to tackle co-occurring conditions in Down syndrome: barriers and facilitators*, chaired by André Strydom (UK). This session provided an overview of transnational research efforts on aspects of Trisomy 21 that require large-scale, representative studies. The workshop opened with the presentation of the DevInDs project (Development of over-inhibition of cortical circuits in Down syndrome), part of the ERA-NET NEURON programme. **Alberto Bacci** and **Marie-Claude Potier** (France) discussed mechanistic studies investigating cortical circuit dysfunction, with a focus on excitation–inhibition imbalance as a neurobiological substrate of cognitive impairment in DS (Zorrilla de San Martin et al., 2020). **Yann Herault** (France) subsequently presented the Go-DS21 programme (ERC Horizon 2020), focusing on gene overdosage and its contribution to comorbidities during early life. The talk emphasized the role of trisomy-driven gene-dosage effects in shaping developmental trajectories and long-term health outcomes, and highlighted translational models designed to dissect genotype–phenotype relationships. The session continued with an overview of the ABC-DS (Alzheimer Biomarkers Consortium–Down Syndrome) initiative, supported by the NIA, presented by **Ben Handen** and **Elizabeth Head** (USA). This large-scale consortium was described as a cornerstone effort to characterize AD biomarkers in adults with DS, integrating neuroimaging, fluid biomarkers, and longitudinal cognitive assessments to define disease staging and facilitate trial readiness. **Rafael de la Torre** (Spain) then presented the ICOD project (Improving Cognition in Down Syndrome), which focuses on a novel pharmacological intervention to enhance cognitive performance in DS. **André Strydom** (UK) also introduced the H21 research network, supported by the Lejeune Foundation, dedicated to advancing collaborative research at the intersection of DS and AD. The network promotes harmonized protocols, shared infrastructures, and coordinated translational efforts across European centers. **Orestes Forlenza** (Brazil) presented the Buriti Network (Rede Buriti), a Brazilian initiative aimed at strengthening research capacity, fostering regional collaboration, and expanding access to clinical and biomarker studies in DS populations across South America. The workshop concluded with a panel discussion that incorporated family organization perspectives, featuring **Bryn Gelaro** (Global Down Syndrome Foundation, USA) and **Marie-Noelle Ungeheuer** (Lejeune Foundation, France). This final segment emphasized the critical role of advocacy groups in shaping research priorities, facilitating participant engagement, and ensuring that scientific programmes align with the needs and expectations of individuals with DS and their families.

## Science & Society

The Science & Society symposium brought together researchers, clinicians, advocates, and individuals with DS to foster dialogue at the intersection of scientific advances and lived experience. The symposium was hybrid, and presentations in English were simultaneously translated into Italian by professional interpreters.

The session opened with an introduction by **Alice Gennaro** (Italy), setting the stage for an afternoon structured around two main thematic blocks: Lifestyle and Health, and Independence and Quality of Life. Chaired by **Lotta Granholm** (USA), the first segment highlighted the importance of preventive and lifestyle-based approaches across the lifespan. **André Strydom** (UK) presented Finger-DS & lifestyle, emphasizing multidomain risk-reduction strategies tailored for individuals with DS. **Yann Herault** (France) discussed the GO-DS study, focusing on gene overdosage and its relationship with early-life comorbidities. **Sujata Bardhan** (USA) introduced the INCLUDE initiative to families, underscoring the role of large-scale, harmonized datasets in advancing precision medicine approaches. **Eimear McGlinchey** (Ireland) addressed the critical issue of raising awareness of DS in the Global South, highlighting disparities in access to care, diagnosis, and participation in research. The session concluded with an open discussion that encouraged interaction between speakers and attendees.

The second thematic block, chaired by **Anna Contardi** (Italy), shifted the focus toward autonomy, empowerment, and quality of life. **Sheri Brynard** (South Africa) delivered a motivational address centered on inclusion and self-determination. A debate with participants with DS, including **Michele Comai** (appointed councillor), **Riccardo Maino** (Italian national FISDIR gymnast and world champion), and **Arianna Sacripante** (synchronized swimming athlete, Italy), provided a powerful platform for self-advocacy and direct engagement, reinforcing the principle that individuals with DS are not only research participants but also active contributors to societal discourse.

The afternoon segment also focused on clinical trials, research participation, and health challenges. A panel on Clinical trials with anti-amyloid therapies and research participation, chaired by **Ann Cohen** (USA) and **Isabel Barroeta** (Spain), addressed both scientific and ethical dimensions of enrolling individuals with DS in AD prevention trials. **Bryn Gelaro** (GLOBAL Down Syndrome Foundation, USA) presented on the role of advocacy organizations in clinical trial outreach, illustrating four case studies and emphasizing strategies to enhance community trust and engagement. **Isabel Barroeta** provided an overview of ongoing anti-amyloid clinical trials in DS, situating them within the broader landscape of biomarker-driven therapeutics. **Annie Cohen** discussed research participation from the perspective of families and community engagement, while the Lumind video participant research study further highlighted innovative approaches to participant-centered research communication. A multidisciplinary debate followed, featuring voices from education, public service, and elite sports, reinforcing the societal breadth of DS advocacy. The session concluded with a dedicated segment on Co-researchers and Health Challenges. **Eric Rubenstein** (USA) presented on the emerging role of co-researchers, individuals with DS actively involved in research design and dissemination, reflecting a paradigm shift toward participatory science. **Sujay Ghosh** (India) discussed pressing health challenges across diverse global contexts, highlighting inequities in healthcare access and the need for culturally sensitive interventions. Closing remarks were delivered by **Anne-Sophie Rebillat** (France) and **María Carmona-Iragui** (Spain), Co-Chairs of the Science & Society Committee of T21RS, who underscored the importance of maintaining a structured, sustained dialogue between science and society as a defining feature of T21RS meetings.

The symposium concluded with closing reflections and a cultural program, reinforcing the central message that scientific progress must advance hand in hand with societal inclusion and meaningful participation.

## Family and Community Engagement

Alongside the scientific sessions, the 5th T21RS International Conference hosted a comprehensive two-day Family Program, conducted entirely in Italian and specifically designed for individuals with DS, their families, and caregivers. This parallel program, a central pillar of the conference’s commitment to societal engagement and inclusivity, involved more than 400 participants from across Italy. Structured around plenary discussions, thematic sessions, and interactive workshops, the program addressed key medical, psychosocial, and life-course issues relevant to people with DS. Topics included physical activity and adaptive sport, affectivity and sexuality, nutrition and feeding difficulties, communication and augmentative strategies, management of medical emergencies, behavioral challenges, transition to adulthood, employment and social inclusion, aging, and dementia risk. Sessions were led by a multidisciplinary faculty comprising clinicians, psychologists, allied health professionals, representatives of national DS associations, policymakers, and employers, ensuring a broad, practice-oriented perspective. A distinctive feature of the program was the active involvement of people with DS themselves, who contributed through experiential narratives on sport, work, relationships, and independent living, reinforcing the value of self-advocacy and lived experience. In addition, dedicated listening points and small-group practical courses facilitated direct interaction between families, healthcare professionals, and researchers, promoting dialogue, empowerment, and the translation of scientific knowledge into everyday care practices. By creating a structured yet inclusive space that placed families and individuals with DS at the center, the Family Program strengthened the bridge between research, clinical care, and community needs, highlighting the essential role of families and self-advocates as partners in shaping research priorities, healthcare pathways, and social policies in DS. The participation of researchers and families was further facilitated by an on-site childcare service during the congress, sponsored by AELIS Farma.

The integration of scientific excellence with societal engagement emerged as a defining feature of the Rome Conference. By actively involving families, self-advocates, clinicians, and researchers within a shared programmatic framework, the meeting strengthened the connection between research priorities and real-world needs. This approach reinforces the role of T21RS as a platform where scientific innovation and community engagement mutually inform each other, setting a model for future international conferences in DS research.

## Awards

### Montserrat Trueta Outstanding Career Award

**Renata Bartesaghi** (Italy), recipient of the Monserrat Trueta Outstanding Career Award for 2024, supported by the Catalan Down Syndrome Foundation, delivered a speech summarizing her career goals and achievements in DS research. Renata Bartesaghi’s studies have focused on the mechanisms underlying brain developmental alterations in DS and on the search for therapies to improve cognitive impairment (Bartesaghi, 2023; Bartesaghi et al., 2022). Her research has shown that trisomy 21 extensively impairs neurogenesis and overall brain development starting from early phases of gestation, which highlights the relevance of the prenatal period as a critical time window for therapeutic interventions. In a mouse model of DS, she provided novel evidence that prenatal/early postnatal pharmacotherapy with the antidepressant fluoxetine can restore neurogenesis, brain development, and cognitive performance. Demonstration that early intervention may counteract the deleterious effects of trisomy 21 on brain development may represent a breakthrough for intellectual disability in DS (Guidi et al., 2014). Her results led to a clinical trial of fluoxetine in children with DS, which showed cognitive benefit. Since making the discovery that fluoxetine positively impacts neurodevelopment in DS, she has tested a variety of drugs in a mouse model to expand the spectrum of possible treatments for people with DS. She found that various treatments, including natural substances, administered at early developmental stages, were effective. The ensemble of her studies has provided proof of principle evidence that early treatment may be a winning card for the prevention of intellectual disability in people with DS.

## Dissertation Awards

T21RS formally recognized the scientific merit and the high-quality research in the field of DS of recently graduated doctoral students by awarding the “Annette Karmiloff-Smith and Michael Harpold Dissertation Awards”. The winners of the “Annette Karmiloff-Smith and Michael Harpold Dissertation Award Program,” launched in 2023 for outstanding PhD theses, were **Jenny Klein** from the Department of Anatomy and Neurobiology, Boston University, Boston, USA, and **Xin Wang** from Wake Forest University School of Medicine, North Carolina, USA. Jenny Klein presented her PhD thesis work entitled “Olig2 neural progenitor cell development and fate in Down syndrome”. Xin Wang presented his dissertation work entitled “Studies on dysregulation of *de novo* protein synthesis in Down syndrome and Alzheimer’s disease”.

## Poster Awards

A poster competition for young investigators was organized, attracting around 130 young investigators. During the poster sessions, a panel of reviewers evaluated the posters based on both the quality of the research content and the authors’ ability to present their research work. Poster awards were assigned during the conference to the following six winners: **Anna Nathanson** from the Broad Institute of MIT And Harvard, Cambridge, MA, USA, for her work “Identifying histone post-translational modifications driving global molecular dysregulation in Down syndrome”; **Natalie Edwards** from Columbia University, New York, USA, for her work ”Contributions of cerebrovascular disease, neuroinflammation, and plasma biomarker concentration to incident Alzheimer’s-related diagnosis in adults with Down syndrome”; **Barbara Zulli** from Sapienza University of Rome, Italy, for her work:” Dipeptidyl peptidase-4 (dpp4) activity is associated with accelerated aging in Down syndrome”; **Lucrezia Romana Rolfi** from Sapienza University of Rome, Italy, for her work “Role of miR-802 in brain insulin signaling and its impact on Down syndrome”; **Katja Sandkühler** from Department of Neurology, University Hospital, LMU Munich, Germany for her work: “Theory of mind in children with Down syndrome”; **Laura Reiche** from Neuroregeneration, Department of Neurology, Medical Faculty and University Hospital Düsseldorf, Germany, for her work ”Stabilizing effects of myelin repair drugs on dysbalanced oligodendroglial differentiation upon C21orf91 overexpression”.

## Young Investigator Support and Training

A defining feature of the Trisomy 21 Research Society (T21RS) conferences is the emphasis on inclusivity, training, and engagement with the global DS community. The 5th International Conference in Rome strongly reinforced these values, with initiatives that supported young scientists, fostered collaborations across continents, and brought together families, clinicians, and researchers. Sixty fellowships were awarded to early-career researchers, ensuring broad international participation and enhancing diversity. Childcare awards and a day care facility were introduced to reduce barriers to participation and to support researchers with family responsibilities. Furthermore, dedicated networking events and mentorship sessions offered trainees opportunities to interact with senior leaders in the field, thereby promoting career development.

## Conclusions and Future Perspectives

The 5th International Conference of the Trisomy 21 Research Society (T21RS), held in Rome in June 2024 during the Society’s 10th anniversary year, marked the largest and most diverse meeting in the history of T21RS. The Conference brought together nearly 500 scientists from 26 countries and more than 900 attendees overall, reflecting the growing global engagement in DS research. The scientific program highlighted the maturation of DS research into a truly multidisciplinary field, encompassing genomics and epigenetics, neurodevelopment, cognition, neurodegeneration, comorbidities, and therapeutic innovation. Several overarching themes emerged from the meeting. First, the field is moving beyond a purely gene–dosage–centric view of DS, with increasing evidence that epigenetic regulation, chromatin remodeling, and transposable element activity play central roles in DS biology. Second, advances in mechanism-based therapies, including gene therapy approaches, immune modulation, and mitochondrial-targeted interventions, demonstrate the growing feasibility of precision medicine strategies tailored to DS. Third, substantial progress has been made toward trial readiness, with fluid, imaging, and digital biomarkers being validated as robust clinical endpoints, thereby facilitating the design of large-scale interventional studies. Fourth, early-life interventions, including prenatal and childhood therapeutic strategies, show promise in modifying developmental trajectories, while underscoring the importance of careful ethical and safety considerations. Importantly, the Conference reinforced the value of global collaboration as a cornerstone of progress in the field. In parallel, the strong engagement with families and communities, most notably through the two-day Family Program in Rome, which involved more than 400 participants, highlighted the importance of bidirectional dialogue among researchers, clinicians, and those directly affected by DS. Looking ahead, DS research is poised for transformative advances. The integration of multi-omic technologies, high-resolution experimental models, and coordinated international infrastructures will accelerate the identification of disease mechanisms and therapeutic targets. At the same time, the active involvement of families, advocacy organizations, and young investigators ensures that future research will remain inclusive, ethically grounded, and socially impactful. Notably, the meeting emphasized DS not only as a condition of high clinical priority, but also as a unique human model for investigating AD and brain aging, with insights that extend beyond trisomy 21 and inform the broader field of neurodegenerative research. In conclusion, the 5th T21RS International Conference affirmed that DS research has entered a new era characterized by precision, translation, and global cooperation. Building on the momentum generated in Rome, the Society looks forward to the next biennial meeting and to continued collaborative efforts to improve health outcomes and quality of life for individuals with DS worldwide.

## Data Availability

No datasets were generated or analysed during the current study.

## Abbreviations

A$$\beta$$: Amyloid beta
AD: Alzheimer’s disease
ADRD: Alzheimer’s disease and related dementias
APP: Amyloid precursor protein
CAA: Cerebral amyloid angiopathy
CHD: Congenital heart disease
CSF: Cerebrospinal fluid
DS: Down syndrome
DSAD: Down syndrome with Alzheimer’s disease
DSBC: Down Syndrome Biobank Consortium
DSRD: Down Syndrome Regression Disorder
DABNI: Down Alzheimer Barcelona Neuroimaging Initiative
EVs: Extracellular vesicles
GWAS: Genome-wide association study
Hsa21: Human chromosome 21
IFNR: Interferon receptor
iPSC: Induced pluripotent stem cell
NDEVs: Neuron-derived extracellular vesicles
OSA: Obstructive sleep apnea
PET: Positron emission tomography
snRNA-seq: Single-nucleus RNA sequencing
T21: Trisomy 21
T21RS: Trisomy 21 Research Society
